# Cancer incidence among the south Asian and non-south Asian population under 30 years of age in Yorkshire, UK

**DOI:** 10.1038/sj.bjc.6605903

**Published:** 2010-09-14

**Authors:** M van Laar, P A McKinney, R C Parslow, A Glaser, S E Kinsey, I J Lewis, S V Picton, M Richards, G Shenton, D Stark, P Norman, R G Feltbower

**Affiliations:** 1 Paediatric Epidemiology Group, Division of Epidemiology, University of Leeds, Leeds LS2 9NL, UK; 2 Department of Paediatric Oncology and Haematology, St James's University Hospital, Beckett Street, Leeds LS9 7TF, UK; 3 Institute of Oncology, Bexley Wing, St James's University Hospital, Beckett Street, Leeds LS9 7TF, UK; 4 School of Geography, University of Leeds, Leeds LS2 9JT, UK

**Keywords:** childhood, adolescent cancer, ethnicity, incidence

## Abstract

**Background:**

Few studies have examined epidemiological differences between ethnic groups for children and young adults with cancer.

**Methods:**

Subjects aged 0–29 years, diagnosed between 1990 and 2005 in the former Yorkshire Regional Health Authority, were included in the analysis. Ethnicity (south Asian or not) was assigned using name analysis program and Hospital Episode Statistics data. Differences in incidence (per 1 000 000 person-years) rates and trends were analysed using joinpoint and Poisson regression analysis.

**Results:**

Overall cancer incidence was similar for south Asians (12.1, 95% CI: 10.7–13.5; *n*=275) and non-south Asians (12.6, 95% CI: 12.2–13.1; *n*=3259). Annual incidence rates increased significantly by 1.9% per year on average (95% CI: 1.2–2.6%), especially for south Asians (7.0% 95% CI: 4.2–9.9%).

**Conclusion:**

If present trends continue, the higher rate of increase seen among south Asians aged 0–29 years in Yorkshire will result in three times higher cancer incidence than non-south Asians by 2020.

Within Europe and the United Kingdom, several cancer epidemiology studies have shown differences in incidence rates between ethnic groups across all ages (Lane *et al*, 2007; Jack *et al*, 2009; NCIN, 2009). Differences in incidence by ethnic group have also been observed amongst children (0–14 year olds) in the United Kingdom. Excesses of all cancers combined and of leukaemia, acute myeloid leukaemia, lymphoma, germ cell and hepatic cancers has been observed amongst south Asians compared with non-south Asians over the last 20 years (Powell *et al*, 1994; Winter *et al*, 1999; Cummins *et al*, 2001; McKinney *et al*, 2003). However, south Asian children have displayed lower incidence rates of Wilms’ tumours and rhabdomyosarcoma (Stiller *et al*, 1991; Powell *et al*, 1994). Furthermore, the well-described incidence peak for leukaemia amongst 2–6 year olds has been observed later in life for south Asians (5–9 year olds) than for non-south Asians (0–4 year olds) (McKinney *et al*, 2003; McNally and Eden, 2004). In the United States, incidence of leukaemia was higher among Hispanics and lower amongst Blacks, compared with White children and adolescents (Bleyer, 2002; Matasar *et al*, 2006). Although the descriptive epidemiology of teenage and young adult cancer in the United Kingdom has been the subject of recent studies, none have focused on differences by ethnic group (Birch *et al*, 2003, 2008). Moreover, it is unclear whether incidence differences exist exclusively in the older adult population or whether they begin to emerge in adolescence or young adulthood.

We have investigated for the first time in the United Kingdom incidence trends in Yorkshire by ethnic group (south Asian or not) across the childhood (0–14 years) and young adult (15–29 years) age ranges, exploiting the availability of population-based data and using information on ethnic group derived and validated using a combination of two name analysis programs and linked hospital episode statistics (HES) data.

## Materials and methods

The data used for this study were extracted from the Yorkshire Specialist Register of Cancer in Children and Young People (YSRCCYP), a population-based data set of children (aged 0–14 years) and young adults (aged 15–29 years) diagnosed with cancer while residing in the former Yorkshire Regional Health Authority since 1974 (Feltbower *et al*, 2009). Patients were eligible if diagnosed between 1990 and 2005 and aged between 0 and 29 years. Skin carcinomas and melanomas were excluded. Case details were ascertained from hospital clinics and neuropathology departments across the region, and additional ascertainment checks undertaken for completeness were carried out with the National Registry of Childhood Tumours (www.ccrg.ox.ac.uk) and the Northern and Yorkshire Cancer Registry and Information Service (www.nycris.org.uk). A total of 85% of all diagnoses recorded on the YSRCCYP have been histologically verified (Feltbower *et al*, 2009).

Tumour diagnoses were categorised into histological groups according to the International Classification of Childhood Cancer (ICCC) (Kramárová *et al*, 1996). For the purposes of certain analyses, malignancies were also grouped into the following four categories to retain statistical power: leukaemia, lymphoma, central nervous system (CNS) tumours and other solid tumours, corresponding to ICCC codes I, II, III and IV–XII.

The former Yorkshire region contained a population of ∼1.4 million 0–29 year olds at the time of the 2001 census, 8% of who were of south Asian ethnic origin. This was an increase of 1.5% in the south Asian population since 1991. In 2001, the study area contained 11% of all 0- to 29-year-old south Asians in England, compared with only 7.4% of all 0- to 29-year-old non-south Asians.

Historically, in the United Kingdom, cancer registry data on ethnic group has not been routinely or accurately, collected and thus alternative methods have been proposed to allocate ethnicity. Ethnicity was assigned as either south Asian (i.e., of Pakistani, Indian or Bangladeshi origin) or non-south Asian (all other ethnicities) initially using the south Asian names analysis program Nam Pehchan and the South Asian Name and Group Recognition Algorithm (SANGRA), with visual inspection by local experts in case of any discrepancy. Both programs have a high level of accuracy and Nam Pehchan is particularly suited to the south Asian population of Yorkshire (Cummins *et al*, 1999; Nanchahal *et al*, 2001).

In addition to both name analysis programs, independent crosschecking was completed using ethnicity data available from linked in-patient HES data. The HES records were matched through NHS number, date of birth, postcode and sex. A total of 62% (*N*=2195) of registered patients were successfully linked to at least one HES record and no ethnic group was recorded in HES for 24% (*N*=517) of the linked patients; thus, 1678 (47.5%) patients’ ethnic classification derived from Nam Pehchan and/or SANGRA included in the analysis were validated using HES. For every hospital episode, ethnicity details are collected separately, which can lead to an individual being allocated multiple ethnicity codes. The most frequently recorded ethnicity code was used to overcome this issue, if present. Ethnicity codes within HES are based on the 16 categories, as used in the 2001 census. These groups were aggregated to a binary classification of south Asian and non-south Asian. In cases in which the most common code was listed as ‘unknown’, the most commonly occurring known group was used instead.

Where clear differences existed between the original 16-category HES code and the name analysis (e.g. White British *vs* south Asian), HES superseded the name analysis classification and the data were changed accordingly. However, in cases in which the HES ethnicity code was ambiguous, such as ‘any other Asian’ or ‘any other ethnic group’, compared with a name classification of south Asian, the latter ethnicity assignment was retained. There was disagreement between assigned ethnicity based on HES data and name analysis in just 2.4% (*n*=40) of cases, of which 23 were re-allocated.

### Statistical analysis

Incidence rates and trends were examined overall and by major histological subtype. Internally, age- and sex-standardised incidence rates (per 1 000 000 person-years) and 95% confidence intervals by ethnic group were calculated using mid-year population estimates of the former Yorkshire Regional Health Authority based on the 1991 and 2001 UK census available by single year of age, sex and ethnic group. Population estimates used between 1990 and 2001 incorporated non-responses that were not included in the original UK census estimates; these figures also take into account changes in census geographies over time (Norman *et al*, 2008; Sabater and Simpson, 2009). Mid-year population estimates from 2002 to 2005 were experimental estimates by ethnic group provided by the Office for National Statistics (ONS, 2001 Census).

Incidence rates were calculated by 5-year age band (0–4, 5–9, 10–14, 15–19, 20–24 and 25–29 years), diagnostic group and gender. Incidence trends were assessed by deriving smoothed 3-year moving averages (MA) for the study period in order to highlight temporal patterns within the data. Initially, joinpoint regression was used to compare differences in the incidence of cancer between south Asian and non-south Asian children and young people, while adjusting for sex, age and year of diagnosis. Poisson regression models were used when joinpoint analysis provided no evidence against a constant average annual percentage change (AAPC) in incidence rates. An interaction term between year and ethnic group was included in the best fitting model to assess whether the effect of ethnic group changed significantly over time. Incidence trends by ethnicity were forecasted up to 2020 by extrapolating predicted values from the final model. Statistical analyses were implemented mostly in Stata 11 (StataCorp, 2009); joinpoint regression was performed using the Joinpoint Regression Program version 3.4.3 (National Cancer Institute, 2010).

## Results

A total of 3534 children and young adults were registered with a tumour between 1990 and 2005 while living in the former Yorkshire region, of whom 275 (7.8%) were south Asian.

The number of cases, comparative frequencies and age- and sex-standardised incidence rates by diagnostic group, age group and gender for south and non-south Asians are given in Table 1. The distribution of cases was very similar across ethnic groups, except for a higher frequency of 25- to 29-year-old non-south Asian cases compared with south Asians. There was a notable excess of male cases compared with females, which was similar across ethnic groups. The incidence rate (per 1 000 000 person-years) for all cancers in 0–29 year olds between 1990 and 2005 was 12.6 (95% CI: 12.2–13.0) and was similar overall between ethnic groups. Rates were non-significantly higher for south Asians with leukaemia and lymphoma, but were lower for CNS and other solid tumours. Slightly higher rates for all cancers combined were observed in south Asian for those aged 5–9, 10–14 and 15–19 years; however, this was not significant. There was no significant difference in incidence when comparing male and female south Asians and non-south Asians for all tumour groups combined.

Age-specific incidence rates by ethnic group for each diagnostic group are given in figure A of the Supplementary Material. A small deficit amongst 25- to 29-year-old south Asians compared with non-south Asians was seen consistently across all diagnostic groups, and was most evident amongst lymphomas and CNS tumours.

Poisson regression models showed that there was a significant difference between the incidence rates of south and non-south Asians by diagnostic group (likelihood-ratio test for ethnic group and diagnostic group interaction; *P*=0.01), adjusting for age group, sex and year of diagnosis. No significant difference in incidence between age group and ethnicity was apparent when testing for an interaction between the two variables.

**Figure 1:**
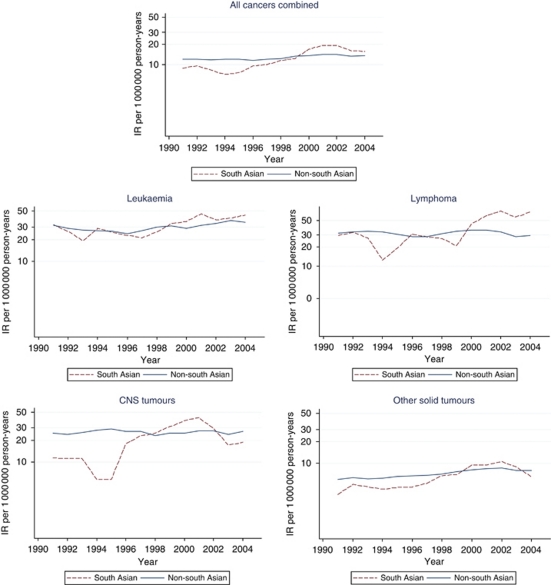
Smoothed (3-year moving average) age- and sex-standardised incidence rates (IR) by ethnic group in Yorkshire, UK (1990–2005).

Figure 1 displays incidence trends between 1990 and 2005 for all cancers combined and for each diagnostic group using smoothed 3-year MA. Joinpoint regression analysis revealed that the best fitting model for all cancers combined and for each diagnostic group was one with no joinpoints; therefore, Poisson regression was used to calculate the AAPC in each case. Overall, there was a significant AAPC of 1.9% (95% CI: 1.2–2.6% *P*-value <0.001). A significant change in incidence was also seen for leukaemia (AAPC=1.8% 95% CI: 0.2–3.4% *P*-value=0.027) and other solid tumours (AAPC=2.9% 95% CI: 1.7–4.1% *P*-value <0.001). The latter change in incidence was accounted for by rises among bone tumours (4.3% *n*=151), germ cell neoplasms (4.4% *n*=614) and carcinomas (5% *n*=94). No significant change in incidence was apparent for lymphoma (1.1%) or CNS (1.0%) tumours.

Likelihood-ratio tests showed significant differences between the temporal change in incidence by ethnic group (an interaction between year and ethnicity) for all cancers combined (*P*=0.0002). Joinpoint regression analysis again showed that the best fitting model in each case for both south and non-south Asians had no joinpoints, and thus the AAPC was calculated using Poisson regression. The rate of increase among south Asians was much higher (AAPC=7% 95% CI: 4.2–9.9%) compared with non-south Asians (AAPC 1.5% 95% CI: 0.8–2.3%); this finding was even more pronounced for the south Asians aged 15–29 years (AAPC=11% 95% CI: 4.0–18.0%). Similarly, there was a significantly higher rate of increase for south Asians with lymphoma (AAPC 12.0% 95% CI: 5–18%) compared with non-south Asians (AAPC 0.1% 95% CI: −1.5 to 1.7%), although the number of south Asians with lymphoma between 1990 and 2000 was extremely small (*n*=28). This meant that rates of all cancers combined among south Asians matched those of the indigenous population by 2005, despite exhibiting much lower rates before the mid-1990s (Figure 1). This difference in incidence trends by ethnic group was not mirrored among those with leukaemia, CNS tumours or other solid tumours.

Temporal incidence rates comparing 0–14 and 15–29 year olds by diagnostic group showed a significant increase in incidence rates for 15–29 year olds between 1990 and 2005 for all cancers combined (AAPC=2.9% 95% CI: 2.0–3.9%), leukaemia (AAPC=4.2% 95% CI: 1.3–7.1%) and other solid tumours (AAPC=4.4% 95% CI: 3.0–5.9%). No significant change in incidence was apparent for 0–14 year olds (0.01% for all cancers combined).

**Figure 2:**
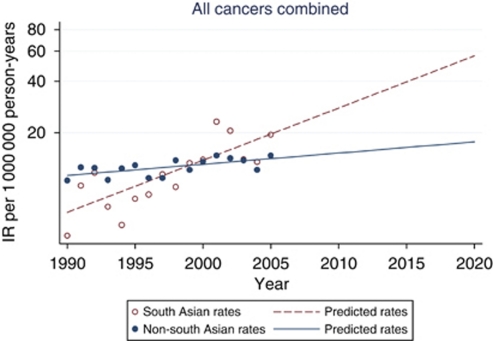
Age- and sex-standardised incidence rates (IR) and predicted rates by ethnic group for all cancers combined.

Figure 2 shows the age- and sex-standardised incidence rates for south and non-south Asians for all cancers combined alongside predicted incidence rates from 2005 to 2020. If the AAPC continues to increase at a constant rate, the incidence of cancer amongst south Asians is set to increase by almost threefold from 2005 onwards to be ∼56 per million compared with 17 per million for non-south Asians by 2020.

## Discussion

We report details of the first population-based epidemiological study looking at incidence rates and trends across the childhood, teenage and young adult age range using a validated ethnic group classification. Previous epidemiological studies focusing on ethnic minority populations have relied on a single source of data on ethnic group (Cummins *et al*, 2001; McKinney *et al*, 2003). However, our analysis incorporated information from the patient's name alongside a self-reported ethnic classification derived from routine hospital admissions. Although we were able to validate only half of the cohort using this data source, misclassification rates were extremely low, suggesting that the primary methodology based on name recognition software algorithms was an accurate and reliable approach. An estimated 60% of the south Asian population within the study region (mainly West Yorkshire) originates from Mirpur in rural Pakistan, making it one of the few regions in the United Kingdom that allows for a detailed analysis of a relatively homogeneous south Asian population (Turton and Gonzáles, 1999; The Change Institute, Department for Communities and Local Government, 2009). We used mid-year population estimates broken down by age, sex and crucially by ethnic group. These were derived from recent research to enhance previous census and mid-year estimates with adjustments for differential non-response by age–sex and ethnic group and on the most recent ONS estimates. We are therefore confident in the accuracy of our population denominator and ethnic group assignment.

We found a significantly higher rate of increase in incidence among the south Asian population of Yorkshire in comparison with non-south Asians, which resulted in similar overall incidence rates between ethnic groups across the study period (1990–2005). This difference in incidence trends was apparent for all cancers combined, but most pronounced for lymphoma, and seen in particular among 15–29 year olds.

Our findings suggest that there may be an aetiological component, possibly environmental in nature, which could be specific to the context of the south Asian population in Yorkshire. Other epidemiological studies focusing on chronic disease epidemiology in Yorkshire have suggested that there may be a dietary (Edwards *et al*, 2006) or infectious (Parslow *et al*, 2001; Feltbower *et al*, 2005) component to aetiology and future work should look in more detail, especially within the lymphoma group, to identify plausible causal hypotheses. The temporal variation in incidence trends by ethnic group could, in part, be confounded by socioeconomic status (Varghese *et al*, 1996) or genetic differences between Mirpuri south Asians and other ethnic groups (De Vos *et al*, 2006). We plan to examine ethnicity with respect to socioeconomic status in a further detailed ecological analysis.

The differential increase in incidence among the south Asian population also has implications for healthcare resource planners in the region. Our incidence projections suggest the burden of disease for south Asians is likely to result in almost three times higher rates by 2020, assuming that the linear increase since 1990 continues and the age structure of the Yorkshire population remains the same over time. The highest proportion of south Asians reside within the highly urbanised district of Bradford in West Yorkshire, comprising 48% of the total south Asian population of Yorkshire, and it is here that the increase in the burden of disease on the NHS will be most strongly felt.

We saw a slightly higher incidence of leukaemia and lymphoma among south Asians, but a deficit for CNS tumours and other solid tumours. This supports findings from previous UK childhood investigations (Stiller *et al*, 1991; Powell *et al*, 1994; Winter *et al*, 1999; Cummins *et al*, 2001; McKinney *et al*, 2003). Studies that looked at the older childhood and young adult age range revealed similar temporal increases in incidence, which was more pronounced among the other solid tumour groups, particularly melanoma and carcinoma (Birch *et al*, 2002; Reedijk *et al*, 2005; Kroll *et al*, 2006). More recently, these changes in incidence could, in part, be attributed to improvements in diagnostic techniques and better case ascertainment, although the latter is unlikely because of annual crosschecks with other regional and national cancer registries and neuropathology databases since the early 1990s (see Materials and Methods), while histological verification of tumours has exceeded 85%.

In summary, despite the similarity in incidence rates between south Asian and non-south Asians in Yorkshire, our data revealed a sharper increase for the south Asian population, which, if present trends persist, will result in a threefold higher incidence by 2020 compared with non-south Asians.

## Figures and Tables

**Table 1:** Number of cases and age–sex standardised incidence rates for south Asians and non-south Asians by diagnostic group, age group and gender (1990–2005)

	**Number of cases**			**Incidence rates**		
	**South Asian, cases (column** **%)**	**Non-south Asian, cases (column %)**	**Total**	**South Asian, rate (95% CI)**	**Non-south Asian, rate (95% CI)**	***P*-value**
*Diagnostic group*						
Leukaemia	66 (24)	649 (20)	715	33.6 (25.4–41.7)	30.3 (27.9–32.6)	0.354
Lymphoma	71 (26)	689 (21)	760	37.8 (28.9–46.6)	31.9 (29.5–34.3)	0.074
CNS tumours	37 (13)	562 (17)	599	19.4 (13.1–25.7)	26.1 (24.0–2.83)	0.111
Other solid cancers	101 (37)	1359 (42)	1460	6.1 (4.9–7.3)	7.0 (6.6–7.4)	0.191
						
*Age at diagnosis (years)*						
0–4	66 (24)	650 (20)	716	16.2 (12.3–20.1)	16.1 (14.8–17.3)	0.918
5–9	40 (15)	364 (11)	404	10.5 (7.2–13.8)	8.5 (7.7–9.4)	0.225
10–14	37 (13)	375 (12)	412	10.5 (7.1–13.9)	8.7 (7.9–9.6)	0.28
15–19	42 (15)	463 (14)	505	11.3 (7.9–14.7)	10.9 (9.9–11.9)	0.723
20–24	41 (15)	597 (18)	638	10.4 (7.2–13.6)	13.4 (12.3–14.5)	0.181
25–29	49 (18)	810 (25)	859	14.2 (10.2–18.2)	18.1 (16.8–19.3)	0.146
						
*Gender*						
Male	159 (58)	1995 (61)	2154	14.2 (12.0–16.4)	15.3 (14.6–15.9)	0.555
Female	116 (42)	1264 (39)	1380	10.0 (8.2–11.8)	9.9 (9.4–10.5)	0.744
Total	275	3259	3534	12.1 (10.7–13.5)	12.6 (12.2–13.1)	0.801

Abbreviations: CI=confidence interval; CNS=central nervous system.
