# Supplementary figures and images for: Cancer incidence among the south Asian and non-south Asian population under 30 years of age in Yorkshire, UK
Source: Br J Cancer. 2010 Sep 14;103(9):1448–52. doi: 10.1038/sj.bjc.6605903 (PMC2990599; doi:10.1038/sj.bjc.6605903)

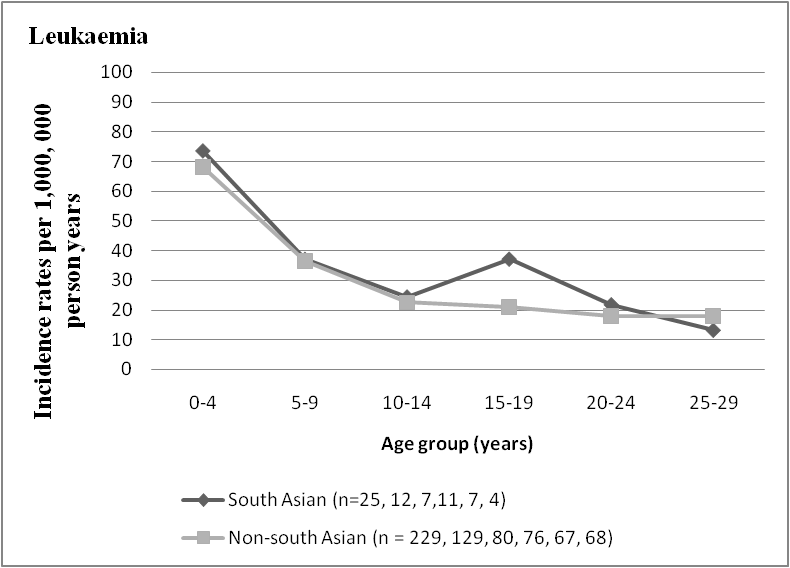

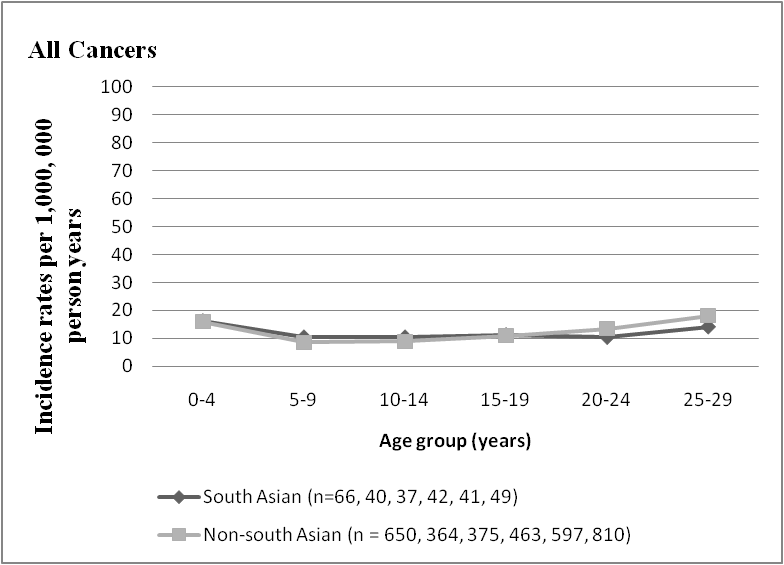

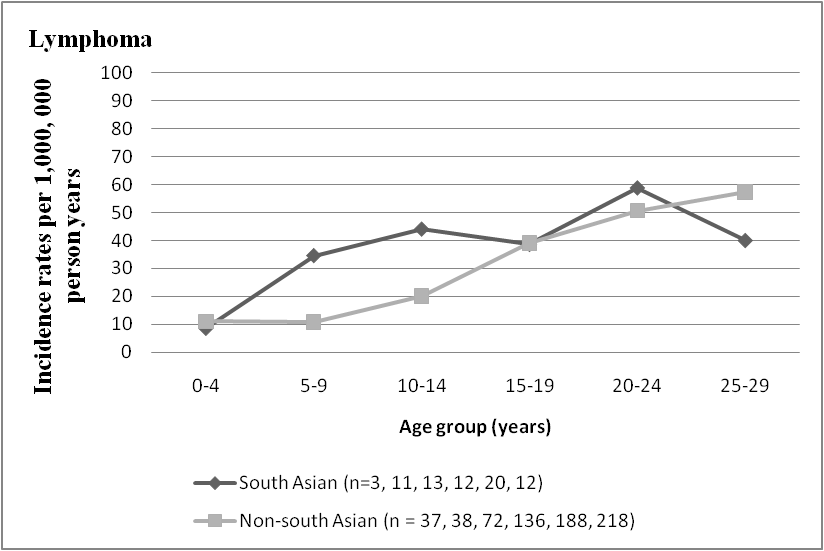

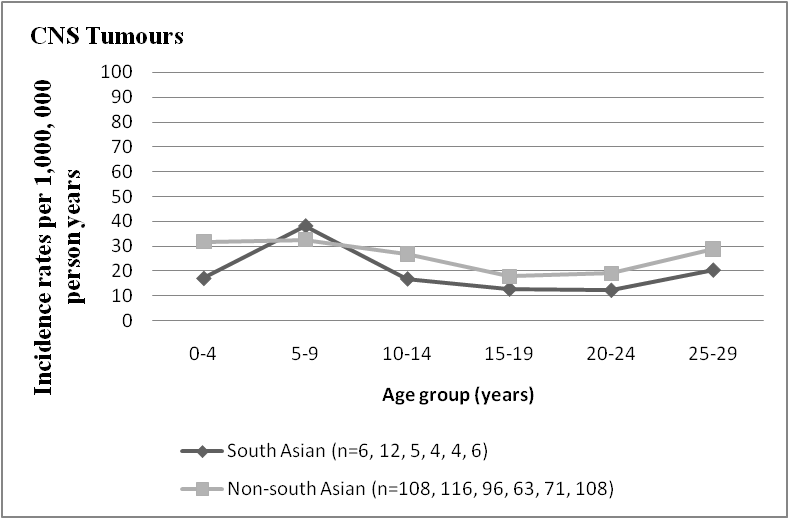

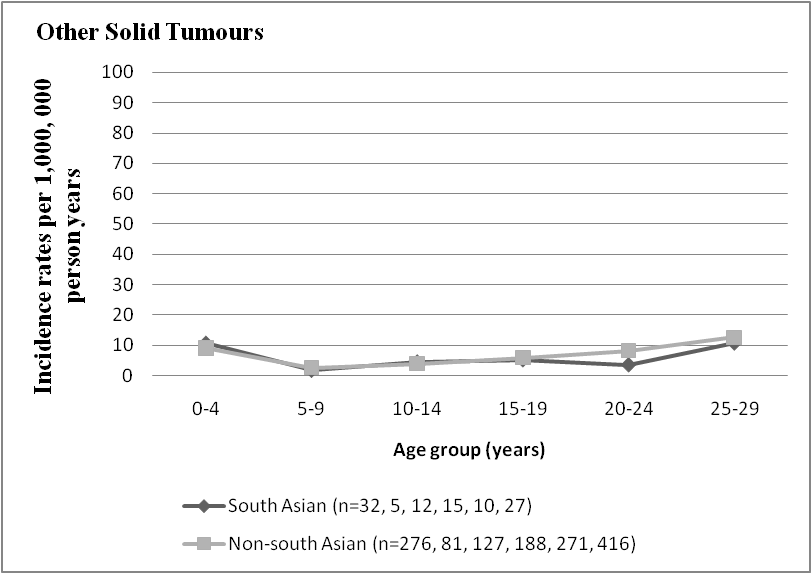

Supplement: Supplementary Material [file 6605903x1.doc]
